# Synthesis and Characterization of Complex Nanostructured Thin Films Based on Titanium for Industrial Applications

**DOI:** 10.3390/ma13020399

**Published:** 2020-01-15

**Authors:** Rodica Vladoiu, Aurelia Mandes, Virginia Dinca, Maria Balasoiu, Dmytro Soloviov, Vitalii Turchenko

**Affiliations:** 1 Faculty of Applied Sciences and Engineering, Ovidius University, 900527 Constanţa, Romania; rvladoiu@univ-ovidius.ro (R.V.); amandes@univovidius.ro (A.M.); 2 Joint Institute of Nuclear Research, Dubna, 141980 Moscow Region, Russia; masha.balasoiu@gmail.com (M.B.); soloviov@jinr.ru (D.S.); turchenko@jinr.ru (V.T.); 3 Horia Hulubei National Institute for Physics and Nuclear Engineering, 077125 Bucharest, Romania; 4 Moscow Institute of Physics and Technology, 141701 Dolgoprudny, Russia; 5 Institute for Safety Problems of Nuclear Power Plants, NAS of Ukraine, 07270 Kyiv, Ukraine; 6 Donetsk Institute of Physics and Engineering named after O.O. Galkin, 83114 Donetsk, Ukraine

**Keywords:** Ti-based layers, TVA deposition method, AFM, SEM, TEM, XRD, SANS

## Abstract

Titanium-based composites—titanium and silver (TiAg) and titanium and carbon (TiC)—were synthesized by the Thermionic Vacuum Arc (TVA) method on substrates especially for gear wheels and camshaft coating as mechanical components of irrigation pumps. The films were characterized by surface morphology, microstructure, and roughness through X-ray Diffraction (XRD), Atomic Force Microscopy (AFM), Scanning Electron Microscopy (SEM), Transmission Electron Microscopy (TEM), and Small-Angle Neutron Scattering (SANS). The silver (Ag) films crystallized into a cubic system with lattice a = 4.0833 Å at room temperature, indexed as cubic Ag group Fm3m. The crystallites were oriented in the [111] direction, and mean grain size was <D>_111_ = 265 Å. The TiC structure revealed a predominant cubic TiC phase, with a = 0.4098 as a lattice parameter determined by Cohen’s method. Average roughness (Ra) was 8 nm for the as-grown 170 nm thick TiAg film, and 1.8 nm for the as-grown 120 nm thick TiC film. Characteristic SANS contribution was detected from the TiAg layer deposited on a substrate of high-quality stainless steel with 0.45% carbon (OLC45) in the range of 0.015 Å^−1^ ≤ Q ≤ 0.4 Å^−1^, revealing the presence of sharp surfaces and an averaged triaxial ellipsoidal core-shell object.

## 1. Introduction

Titanium, with the highest strength-to-density ratio of any metallic element, has outstanding properties that have led to its widespread use in several important technological applications. For instance, due to its high corrosion resistance, titanium is used as bulk or coating in propeller shafts, rigging, and other parts of boats that are exposed to seawater or a salty environment [1]. Resistance to high temperatures allows titanium and titanium alloys to be used in airplanes, missiles, and rockets, and thanks to its excellent biocompatibility, there is great demand for it in artificial hips, pins for setting bones, and for other biological implants [2,3,4].

Silver has also been recognized as a well-matched biomaterial, mechanically and chemically stable, and various techniques can be applied to modify its surface properties. Moreover, in wear conditions, the incorporation of silver (Ag) into titanium (Ti) compounds can change their properties by acting as a solid lubricant [5,6,7]. Ti embedded in a carbon (C) matrix could improve adherence of the coated substrates to obtain better hardness and anticorrosive properties [8,9].

However, one of the main drawbacks of titanium is the difficulty in producing a reliable and strong joint due to a lack of metallurgical compatibility and the formation of intermetallic compounds (IMCs) between the two materials. This shortcoming can be surpassed by using Ti–silver or Ti–carbon composites, thereby producing a highly reliable joint structure [10].

Many methods, such as magnetron sputtering [11], chemical vapor deposition (CVD), and electric discharged plasma, have been studied concerning these drawbacks. Applying vacuum arc techniques to this field has still rarely been reported, as has combining all these properties. In this study, we synthesized titanium-based nanocomposite layers—titanium and silver (TiAg) and titanium and carbon (TiC)—on glass, on a silicon (Si) wafer, and a special substrate with a great interest for industrial applications, OLC45—meaning high-quality stainless steel with 0.45% carbon—using the Thermionic Vacuum Arc (TVA) method. This procedure combines the advantages associated with vacuum techniques and the relatively high rate of deposition, especially ease in tailoring film composition by controlling input arc power [12,13,14].

The aim of this work was to investigate the behavior of composite films that could allow their optimization for advanced engineering applications by using TVA technology, especially for gear wheels and camshaft coating as mechanical components of irrigation pumps.

## 2. Experiment Details

### 2.1. Synthesis of Ti-Based Nanocomposite Films

The samples were synthesized using a Ti-99.99% and Ag-99.9% metal basis, and C-99.99% purity grains provided from the Alfa Aesar company (Lancashire, UK). Samples were prepared on glass. One side had polished crystalline-silicon (c-Si) substrates with a size of 1 × 1 cm^2^ immersed in ultrasonic cleaner provided from Cole–Palmer (Vernon Hills, IL, USA) with a highly effective special cleaner (ultrasonol) for 10 min, and then rinsed with technical-grade acetone for rapid drying. Another substrate demanded by one industrial partner was OLC45, a round-shaped disc (Φ = 15 mm), polished according to the requirements of its application and cleaned as described before. The substrates were mounted on the holder and loaded into the preparation chamber.

**Figure 1:**
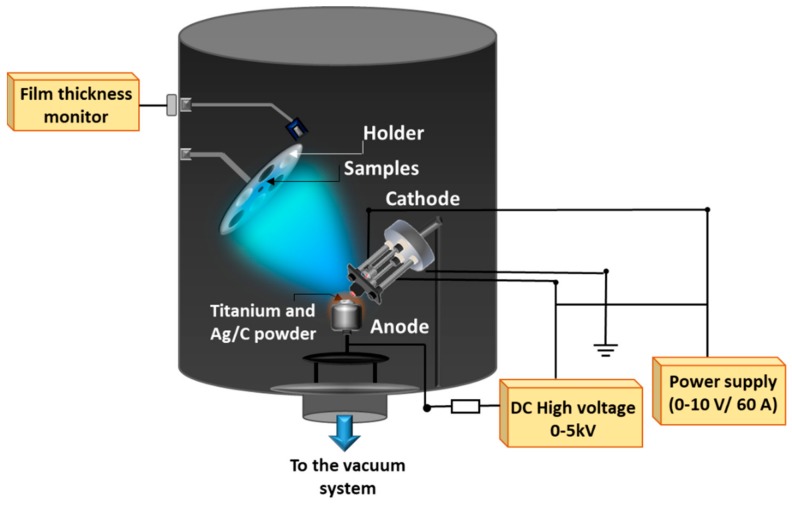
Titanium-based (Ti-based) deposition with basic elements of Thermionic Vacuum Arc (TVA) setup.

Figure 1 shows the experiment setup during Ti–Ag deposition by the TVA method. The thermoelectrons generated from the heated cathode, a tungsten filament mounted inside a Wehnelt cylinder—are focused toward the anode surface. The anode, a crucible filled with the grains of material to be evaporated, was kept at a high positive voltage. Next, the grains of materials (Ti/Ag or Ti/C in our case) were evaporated; therefore, a steady-state density of vapors was established in the interelectrodic space [15]. Applied high DC voltage of up to 5 kV between the electrodes accelerated the electrons coming from the filament and, likewise, vapors from the anode were ionized. This type of deposition ensured high-quality films with a comparatively high deposition rate due to the bombardment of the growing layer by energetic ions of the same material in vacuum conditions. Detailed TVA setup was previously reported [16,17].

The main experiment parameters of this study are listed in Table 1. Base pressure (p_B_) means the residual pressure in the vacuum chamber before heating the filament. Working pressure (p_w_) was pressure during deposition of the thin films. The other parameters are: d_A-S_, distance between samples and discharge ignition point; d_A-C_, distance between anode and cathode; and I_F_, current intensity of heating filament. Input arc power (P) was the arc voltage times the intensity of the arc’s current during deposition [18,19]. The thicknesses (t) of the deposited films were recorded in situ using the MTM 10 thickness monitor (Cressington Scientific Instruments, Watford, UK).

### 2.2. Characterization Methods

Thin films were investigated using microscopy techniques: Transmission Electron Microscopy (TEM; CM 120 ST, Phillips Electronics, Eindhoven, The Netherlands), acceleration voltage of 120 kV, resolution point of 1.4 Å, and 1.2 million× magnification) together with Atomic Force Microscopy (AFM, Veeco CP-R, New York, NY, USA) and Scanning Electron Microscopy (SEM, Zeiss EVO 50 SEM having LaB_6_ cathode, Carl Zeiss, Oberkochen, Germany). Thin films were investigated using transmission electron microscopy techniques: TEM, selected-area electron diffraction (SAED), and High-Resolution TEM (HRTEM). Furthermore, iTEM software (Radius 2.0 version, Emsis, Muenster, Germany) was used for imaging connected with MegaView III on the CM120ST microscope (Emsis, Muenster, Germany). Samples for TEM investigation were prepared using the scratch method [20] with a diamond knife and formvar-covered grids.

X-ray Diffraction (XRD) measurements were carried out to determine the crystalline structure of the deposited TiAg thin films using an Empyrean XRD diffractometer with parallel-beam geometry of Cu-radiation with a scanning step of ~0.026 degrees. Analysis of X-ray diffraction patterns was performed with the Rietveld method using HighScore Plus software (Version 4.8, Malvern Panalytical B.V., Malvern, UK).

The resolution function of the diffractometer was established with the LaB_6_SRM 656b standard (NIST). The characteristic dimension of coherent scattering regions (D_hkl_) was determined by the Scherrer equation:

(1) $$D_{\left(hkl\right)}=\frac{K\lambda }{\beta \cos\Theta },$$

where *D*_(*hkl*)_, characteristic dimension of coherent scattering regions; *K*, Scherrer constant; *λ*, wavelength of X-ray radiation; *β*, integral width at half maximum; and Θ, Bragg reflection angle [21].

Small-angle neutron-scattering (SANS) experiments consist of measuring the intensity of the scattered neutrons versus the amplitude of the scattering wavevector defined by *Q* = 4π/*λ*sin(θ/2), where θ is the scattering angle and *λ* is the neutron wavelength.

SANS measurements were performed on the time-of-flight YuMO spectrometer (JINR, Dubna, Russia) with two detector modes [22] in function at the IBR-2 high-flux pulsed reactor (JINR Dubna). The SONIX^+^ software system (Version 2017, JINR, Dubna, Russia) controlled the spectrometer [23]. Experiments were carried out at a sample-to-detector distance of 5.28 and 13.04 m, resulting in a Q range of 0.006–0.06 A^−1^. Sample diameter and thickness in the beam were 14 and 2 mm, respectively.

For a microstructure comprising a particulate morphology that is randomly oriented (isotropic), the small-angle neutron-scattering intensity can be expressed as:

(2) $$I\left(Q\right)=n{\left|\Delta \rho \right|}^{2}V^{2}P\left(Q\right)S\left(Q\right),$$

where *n,* particle-number density, each with volume *V*; |Δ*ρ*|, scattering contrast; *P*(*Q*), scattering form-factor term for individual particles; and *S*(*Q*), effective structure factor for the arrangement of particles within the sampling volume. Scattering contrast |Δ*ρ*| is the difference between the neutron-scattering length density of the scattering particles (in our case, particles, voids, or pores) and that of the surrounding medium. The form- and structure-factor functions were derived to enable the small-angle scattering from a wide range of heterogeneous morphologies to be described and quantified.

## 3. Results and Discussion

### 3.1. Microscopy Techniques

#### 3.1.1. Atomic Force Microscopy (AFM)

The surface morphology and roughness of the titanium-based films were inspected by AFM under the tapping mode with a silicon cantilever.

**Figure 2:**
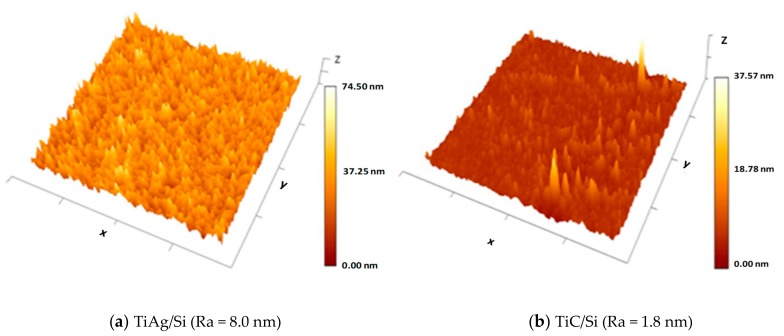
Atomic Force Microscopy (AFM) images of titanium-based nanocomposite layers (**a**) TiAg and (**b**) TiC layers on silicon substrate.

Figure 2 shows 3D AFM images of the TiAg and TiC films on the one-side polished silicon substrate. Surface morphology was different between the two samples (4 µm × 4 µm) deposited on the same substrate. In the case of TiAg/Si (Figure 2a), there were many randomly distributed peaks and valleys, while the surface of the TiC/Si (Figure 2b) was quite uniform, with only a few profile height deviations from the mean line, recorded within the evaluation length. Average roughness (Ra) was 8 nm for the as-grown 170 nm thick TiAg film, and 1.8 nm for the as-grown 120 nm thick TiC film.

#### 3.1.2. Scanning Electron Microscopy (SEM)

**Figure 3:**
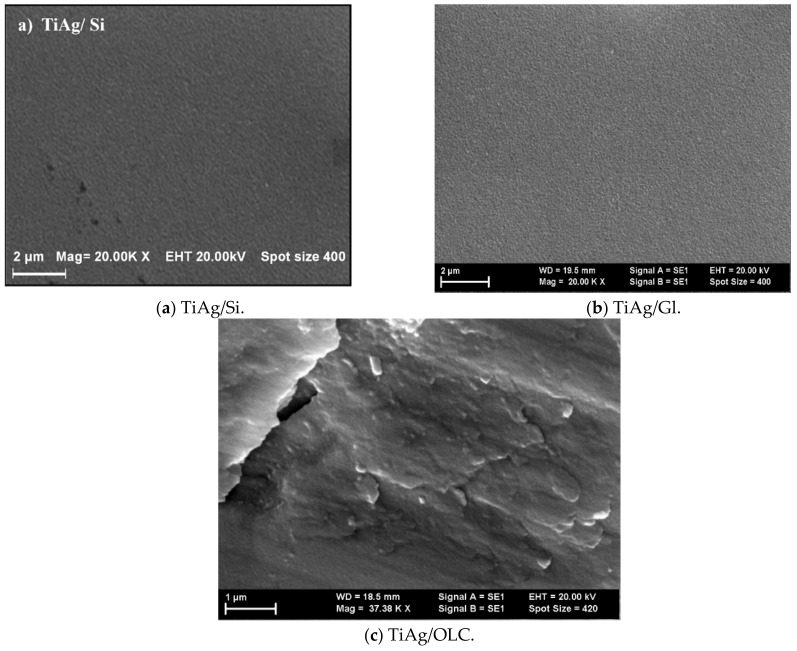
Scanning Electron Microscopy (SEM) micrographs of titanium and silver (TiAg) nanocomposites coating deposited on (**a**) silicone, (**b**) glass and (**c**) substrates of high-quality stainless steel with 0.45% carbon (OLC45).

With 20,000× and 1580× enlargement, as shown in Figure 3, topography of the TiAg nanocomposites deposited on three different substrates was visible. It can be seen that the TiAg film was homogeneously and uniformly coated on the silicon (Si) plates (Figure 3a) and on glass (Figure 3b), without delamination from the substrate. The homogeneity of the coated film surface deposited on a substrate of high-quality stainless steel with 0.45% of carbon (OLC) substrate (Figure 3c) was lower than that on other substrates, but it is an inherent feature of the deposition of Ti-based coating on stainless steel.

The TiAg sample deposited on the OLC substrate was covered by irregular large surfaces with small protuberances distributed on the top of them. Some nanoparticles with a size of 100–200 nm were evenly distributed and embedded in a titanium matrix. This is the typical microstructure of nanocomposite materials. Adherence was improved, producing results in reduced particle size with very fine particles centered at the surface boundaries.

#### 3.1.3. Transmission Electron Microscopy (TEM)

**Figure 4:**
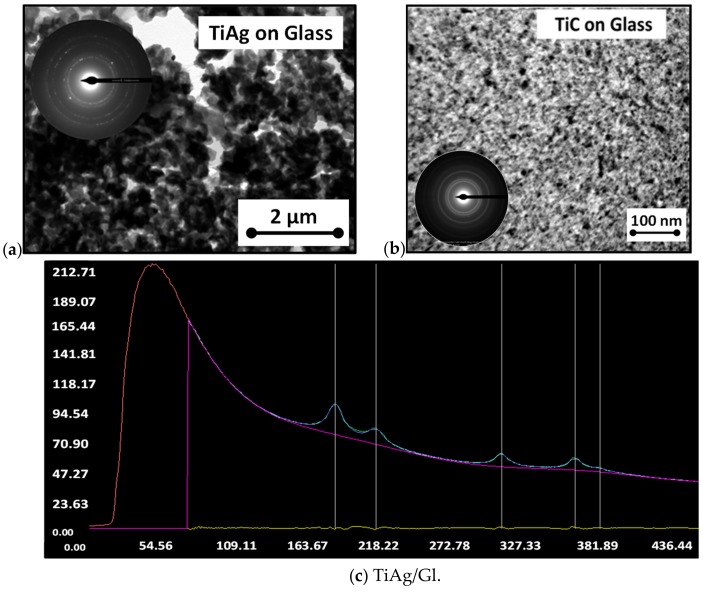

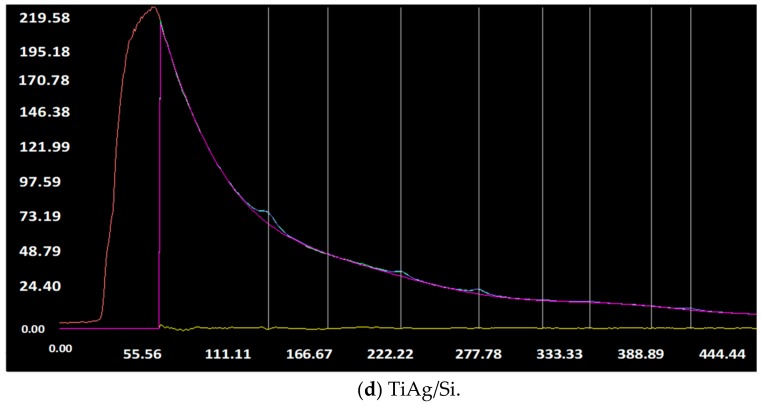
Transmission Electron Microscopy (TEM) images of Ti-based coatings on glass: (**a**) TiAg; (**b**) TiC and profiles from selected-area electron diffraction (SAED) with identified peaks images; (**c**) TiAg on glass; and (**d**) TiAg on silicon.

Detailed microstructure characterizations by TEM of the TiAg and TiC deposited on the glass are presented in Figure 4. Figure 4a shows the bright-field TEM image of the TiAg compound coating. The pattern obtained by selected-area electron diffraction (SAED) was inserted into the image. Electron diffraction was carried out at 880 mm camera length. The corresponding SAED pattern revealed a polycrystalline TiAg that corresponded to the interplanar distance of TiAg (111) plane (d = 0.23642 nm, PDF #65-8470) and Ti phases with the hexagonal structure. The cubic Ag structure was also identified as a major phase in this case.

The Ag structure can also be seen in the HRTEM image of the TiC intermetallic compound coating on glass (Figure 4b) with the predominant cubic TiC phase and lattice parameter determined by Cohen’s method as a = 0.4098 with −5.59% relative error [24]. SAED patterns matched the standards of titanium carbide (TiC), as shown in the insert.

In Figure 4c,d, intensity (in arbitrary units, u.a.) is depicted on the *y*-axis, and the radius, in pixels, on the *x*-axis. Results were converted into degrees and angstroms in a text file saved by the CRISP2 software using a user camera constant (in our case, 44.56 nm × pixel). “Odd” numbers were calculated by CRISP2 on the basis of the radius of the selected circle from the diffraction pattern and intensity maximum (for each axis, 10 numbers are shown).

The obtained data were indexed by comparison with the cubic structure of [TiC] [25] (Table 2). From Figure 4a, it can also be observed that the coating contained nanocrystalline grains, which can be explained by the high energy of ions in the TVA method [26].

SAED profiles were extracted from these patterns using the radial distribution function implemented in CRISP2 software and used to identify structural characteristics [27,28,29]. Using the ELD module from CRISP2, the background was fitted with a 9th degree polynomial function. The automated procedure implemented in ELD was used to identify peaks, but we also applied a manual selection for a peak with low intensity [30]. Results were used to index diffraction peaks and to evaluate lattice parameters. Figure 4c,d presents a snapshot of a CRISP2-integrated profile calculated from the diffraction pattern for TiAg films deposited on glass (Figure 4c) and silicon (Figure 4d).

In the case of TiAg/Gl, electron diffraction showed polycrystalline material characteristics. The peaks evaluated from the profile of the SAED pattern were located as 0.231nm (111), 0.200 nm (200), 0.141 nm (220), 0.121 nm (113), and 0.114 nm (222), and indexed as cubic Ag group Fm−3m, a = 0.4080 nm [25].

As shown in Figure 4d, the TiAg/Si sample had an amorphous character; the major crystalline phase was identified as cubic Si. The Moire fringes found on some particles of the film could be attributed to the fact that the samples were partially crystalline. Using the profile extracted from this pattern, the following values were found: 0.318 nm (111), 0.194 nm (220), 0.158 nm (113), 0.137 nm (400), 0.125 nm (331), and 0.112 nm (224), indexed using cubic structure of Si [27].

These results strongly agree with the XRD investigations.

### 3.2. X-ray Diffraction (XRD) Investigations

**Figure 5:**
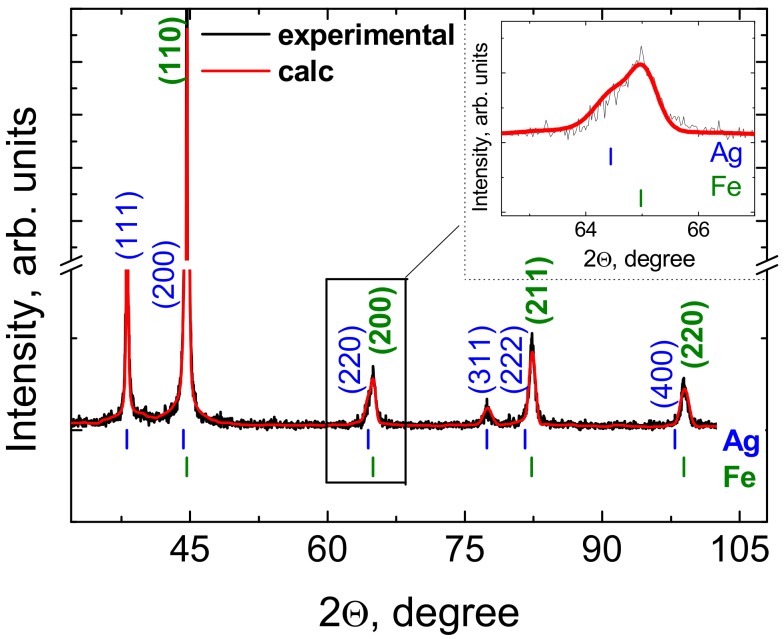
X-ray Diffraction (XRD) pattern at room temperature of TiAg/OLC45 sample.

The XRD pattern at room temperature of the TiAg deposition on the OLC45 sample is presented in Figure 5. Black solid lines mark the observed data, while the red one represents the calculated curve.

The vertical lines shown below the pattern mark revealed the expected Bragg positions of the model, as follows: upper lines mark the Ag position, and the lower lines mark the position for iron (Fe). Meanings of calculated Bragg positions and their structural compliance with the ICDD reference are shown in Table 3. The full width at half-maximum (FWHM) intensity is also presented, clearly fitting very well. No unexpected peak was present, so the sample was practically single-phase. The Fe target was crystallized into a cubic system with lattice a = 2.8661 Å. The fit was performed using the symmetry of space group Im−3m. The Ag film was crystallized into a cubic system with lattice a = 4.0833 Å at room temperature. The crystallites were oriented in the [111] direction, and mean grain size was <D>_111_=265 Å. The fit was performed using the symmetry of space group Fm−3m. The absence of peaks for Ti in the pattern indicated an amorphous phase.

### 3.3. SANS Investigations

The small-angle neutron-scattering method was applied for the investigation of the TiAg/OLC45 sample. The aim of the SANS investigations was detecting possible forming nanostructures in the layer volume with dimensions in the range of 1–100 nm.

In Figure 5 (black squares), the SANS experiment curve is depicted. Two scattering regions were detected in the measured Q-range: (i) 0.015 Å^−1^ ≤ Q ≤ 0.055 Å^−1^, and (ii) 0.06 Å^−1^ ≤ Q ≤ 0.4 Å^−1^.

#### 3.3.1. 0.015 Å^−1^ ≤ Q ≤ 0.055 Å^−1^

**Figure 6:**
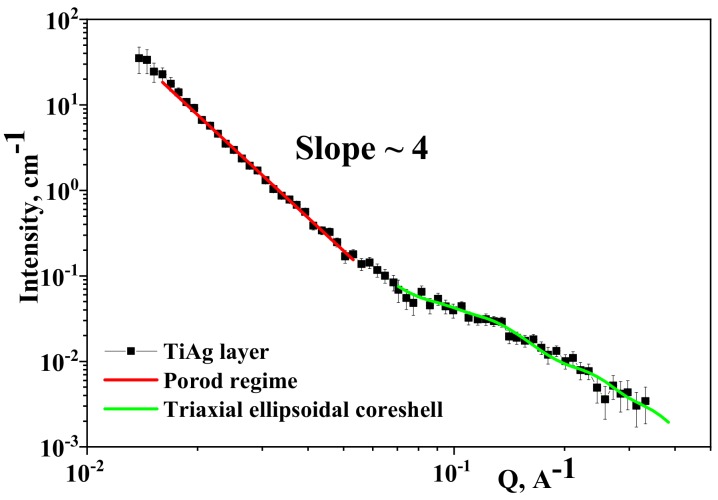
Small-angle neutron scattering (SANS) experiment curve from TiAg/OLC45 sample (black squares); linear fit of curve in 0.015 Å^−1^ ≤ Q ≤ 0.06 Å^−1^ (red line) domain using Porod approximation; model curve fittings with triaxial ellipsoidal core-shell model fit (green line) for experiment data in range of 0.06 Å^−1^ ≤ Q ≤ 0.4 Å^−1.^

In the domain of 0.015 Å^−1^ ≤ Q ≤ 0.055 Å^−1^, the obtained SANS profile manifested a power-law scattering of *I (Q) ≈ Q^−α^* with α = 4 (Figure 6, red line). This case represents the so-called Porod limit and characterizes sharp surfaces/interfaces of the scatterers, for example, the boundary between two regions with different scattering-length densities:

*ρ_Ti_* = −1.9·10^10^ cm^−2^ and *ρ_Ag_* = 3.468·10^10^ cm^−2^,

where *ρ_Ti_* and *ρ_Ag_* represent the scattering length densities of Ti and respectively Ag.

#### 3.3.2. 0.06 Å^−1^ ≤ Q ≤ 0.4 Å^−1^

For SANS data treatment in the range of 0.06 Å^−1^ ≤ Q ≤ 0.4 Å^−1^, a multiparameter fitting method using implemented theoretical models for structural refining was applied through the FITTER program.

The obtained form factor describing the averaged scattering object for the Q-region under consideration was the triaxial ellipsoidal core-shell model (see Figure 6, green fit).

The parameters of the triaxial ellipsoidal core-shell model detected from the experiment curve are displayed in Table 4, where a–c are the half-axis of the core, respectively, and t is shell thickness.

The obtained object was consistent with the model of Ag grains surrounded by a Ti layer or dispersed in a Ti matrix. The Ag grain averaged dimension obtained from XRD agreed with the parameters obtained for the core of the SANS model.

## 4. Conclusions

TiAg and TiC nanocomposites were successfully synthesized on special substrates by the Thermionic Vacuum Arc (TVA) method. The microscopy techniques, together with XRD results, were in very good agreement concerning the structure of TiAg films deposited on glass and OLC: Ag crystallized into a cubic system [111] direction, and mean grain size was <D>_111_ = 265 Å, with lattice a= 4.0833 Å at room temperature. TiAg/Si had an amorphous character, partially crystalline, according to the profile extracted from the pattern. The structured of TiC nanocomposites revealed the predominant cubic TiC phase with the lattice parameter of a = 0.4098 matching the standards of titanium carbide. The roughness of the titanium-based films decreased with the increase of the ions’ energy densities from the plasma core (Ag versus C).

As far as we know, this is the first time that the SANS technique was used for the detection of possible nanoinhomogeneities inside compounds obtained using the TVA method. In the measured scattering curve, characteristic SANS contribution was detected from the TiAg layer deposited on the OLC substrate in the range of 0.015 Å^−1^ ≤ Q ≤ 0.4 Å^−1^, characterizing the presence of sharp surfaces and an averaged triaxial ellipsoidal core-shell object.

These results suggest that the thermionic vacuum arc technology as a deposition process is an efficient method to improve the comprehensive properties of titanium-based films, which act as candidate material for protective coatings, especially for the mechanical components of irrigation pumps.

## Figures and Tables

**Table 1:** Experiment parameters for synthesis of titanium-based nanocomposites.

Parameters	Ti–Ag	Ti–C
Base pressure p_B_ (Pa)	4 × 10^−5^	7 × 10^−5^
Working pressure p_w_ (Pa)	6 × 10^−4^	7 × 10^−4^
Substrates	Si, glass, OLC	Glass
Distance d_A-C_ (m)	5 × 10^−3^	
Distance d_A-S_ (m)	6 × 10^−2^	
Intensity current on filament I_F_ (A)	47	48
Input arc power P (kW)	1.05	1.37
Film thickness t (nm)	120	170
Rate of deposition r (nm/s)	3.5	2.5

**Table 2:** Measured distances corresponding to the peaks compared with the cubic structure of [TiC].

Peak No.	Distance (nm)	hkl	Distance (nm) (TiC)
1	0.24764	111	0.249877
2	0.21596	200	0.216400
3	0.15223	220	0.153018
4	0.12968	113	0.130494
5	0.12424	222	0.124939

**Table 3:** Summaries of phase composition and refined structural parameters, and selected interatomic distances, respectively.

No.	2θ (degr.)	d_hkl_ (Å)	hkl	Rel. Int. (%)	Full Width at Half-Maximum (FWHM, °2θ)	Ref. ICDD PDF No.
1	38.09	2.3610	(111)	26.8	0.270	01-071-3762
2	44.28	2.0440	(200)	6.4	0.391	01-071-3762
3	44.62	2.0291	(110)	100.0	0.331	**01-080-3816**
4	64.44	1.4448	(220)	3.6	0.782	01-071-3762
5	64.98	1.4341	(200)	4.8	0.527	**01-080-3816**
6	77.41	1.2319	(311)	2.2	1.069	01-071-3762
7	81.56	1.1794	(222)	0.8	1.172	01-071-3762
8	82.29	1.1707	(211)	9.3	0.733	**01-080-3816**
9	97.93	1.0212	(400)	0.3	1.646	01-071-3762
10	98.91	1.0137	(220)	4.6	0.989	**01-080-3816**

01-071-3762 (taken from Owen, E.A., Yates, E.L., *J. Chem. Phys.*, 3, 605, (1935)). 01-080-3816 (taken from Crisan, O., Crisan, A.D., *J. Alloys Compd.*, 509, 6522, (2011)).

**Table 4:** Object form factor and its dimensions from SANS experiment-data processing (in Q-range 0.060 Å^−1^ ≤ Q ≤ 0.4 Å^−1^) of TiAg layer (deposited on OLC45 substrate).

Model	Triaxial Ellipsoidal Core-Shell (nm)
Dimensions	a = 15.7 ± 0.1
	b = 14.1 ± 0.1
	c = 6.5 ± 0.1
	t = 4.3 ± 0.1
